# Lamotrigine-induced DRESS Syndrome Manifesting as ‘Eosinophilic Colitis’: An Uncommon Presentation of a Very Uncommon Condition

**DOI:** 10.7759/cureus.7570

**Published:** 2020-04-07

**Authors:** Meghana Parsi, Catherine Daniel

**Affiliations:** 1 Internal Medicine, Crozer-Chester Medical Center, Upland, USA; 2 Internal Medicine, Baylor College of Medicine, Houston, USA

**Keywords:** dress, drug reaction, lamotrigine

## Abstract

Drug reaction with eosinophilia and systemic symptoms (DRESS) is a rare drug-induced hypersensitivity reaction that manifests with a variety of signs and symptoms. It is an important condition that must be recognized by all physicians because if untreated, it can be fatal. There are a variety of medications that are responsible for this condition. The liver, lungs, and kidneys are commonly affected, with the involvement of the gastrointestinal tract being very rare; only a few cases are reported worldwide. We are presenting a case of lamotrigine-induced DRESS syndrome manifesting as colitis. A 32-year-old female presented with diarrhea, two weeks after the initiation of lamotrigine. Her condition worsened with the development of a generalized rash and bloody diarrhea. Further investigations revealed that she likely had a drug reaction secondary to lamotrigine. Fortunately, prompt initiation of systemic steroids led to the resolution of her symptoms.

## Introduction

Drug reaction with eosinophilia and systemic symptoms (DRESS) syndrome is an idiosyncratic drug-induced reaction that mainly affects the liver, lungs, and kidneys. Hepatitis is the most common feature in the majority of these patients. It presents commonly with fever, lymphadenopathy and a variety of other systemic signs. It is important that healthcare providers are aware of this syndrome. If untreated, the mortality rate is between 10-20% [1]. While there are several organs that are commonly involved, we present here a case of lamotrigine, a commonly used anti-seizure medication, induced DRESS syndrome manifesting as colitis. The incidence of DRESS involving the gastrointestinal tract is rarely reported. Thus, this case report helps to demonstrate the various manifestations of DRESS syndrome.

## Case presentation

**Figure 1:**
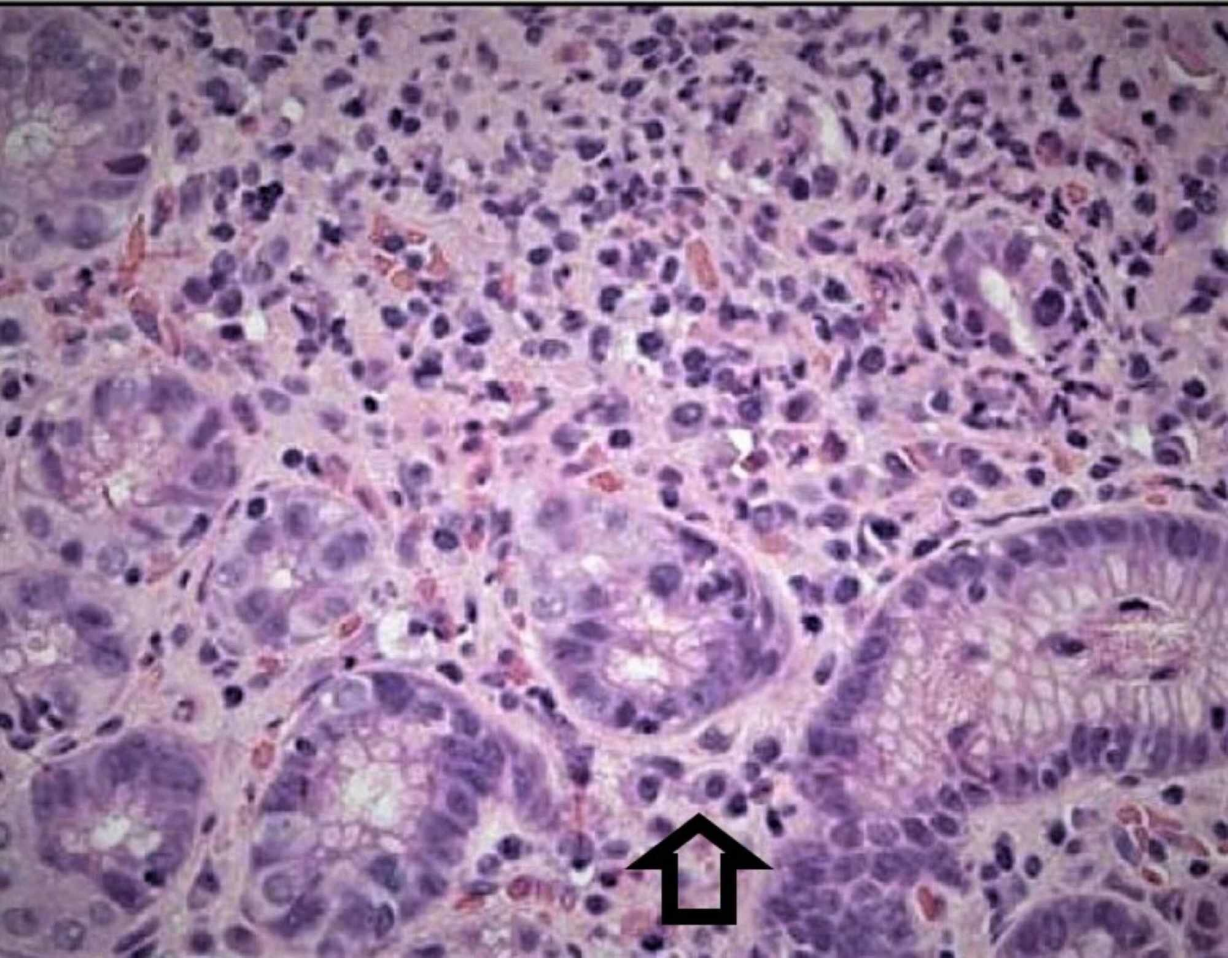
Biopsy, ascending/descending colon Colitis with excess eosinophils in the lamina propria (arrow).

A 32-year-old female with opiate use disorder and bipolar depression, presented with acute onset of abdominal pain and non-bloody diarrhea for the past two days. Two weeks before hospitalization, she was prescribed lamotrigine at a drug rehabilitation center for bipolar disorder. On physical examination, her vital signs were with normal limits. Apart from dry mucous membranes and mild generalized abdominal tenderness, her physical exam was unremarkable. Our initial working diagnosis included opiate withdrawal associated with diarrhea vs viral gastroenteritis. She was managed conservatively with intravenous fluids. However, diarrhea worsened and it became bloody. She subsequently developed fever, facial edema and a red, violaceous maculopapular rash on her arm. An initial concern for invasive bacterial gastroenteritis led to the initiation of intravenous broad-spectrum antibiotics. Unfortunately, her symptoms worsened and the rash spread to more than 50% of her body. Labs were significant for leukocytosis (17 x 10^9^/L, reference range: >11 x 10^9^/L), eosinophilia (43%, reference range: <6%) and atypical lymphocytosis (18%, reference range <5%). Her liver function tests (LFTs) were within normal limits. The remaining blood work was normal including chemistry and thyroid profile. Stool studies were negative for invasive pathogens, including parasites. Given the unrelenting bloody diarrhea, a colonoscopy with biopsy was done and this showed pancolitis with prominent eosinophilic infiltrates (Figure 1).

**Figure 2:**
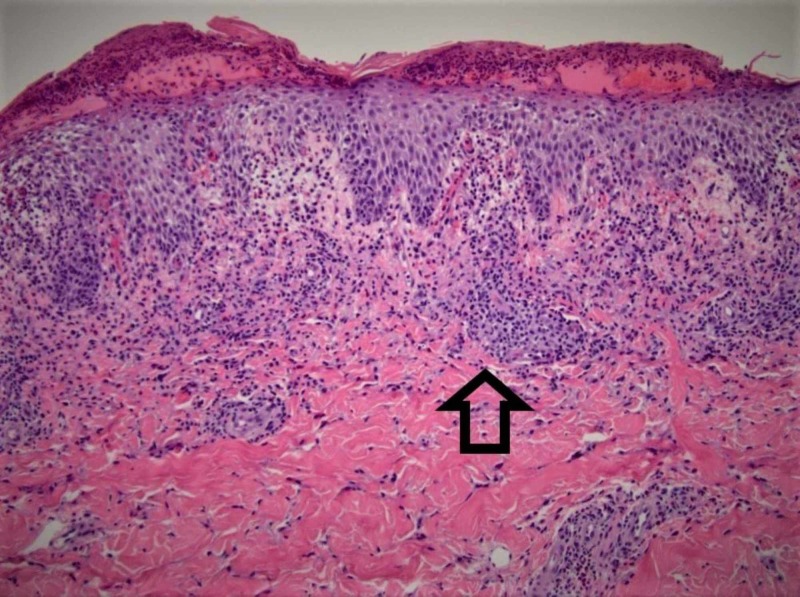
Biopsy, skin Mild spongiosis, vacuolar interface dermatitis, superficial and deep perivascular lymphocytic infiltrate with eosinophils (arrow).

A skin biopsy was undertaken to assess the rash and this revealed vacuolar interface dermatitis, perivascular lymphocytic infiltrate with eosinophils, consistent with a morbilliform rash (Figure 2).

A diagnosis of DRESS syndrome was made based on a “regiSCAR” score of 6. The drug lamotrigine was presumed to be the cause of her symptoms and was stopped. She received a course of systemic steroids after which her diarrhea, rash, and eosinophilia resolved.

## Discussion

DRESS syndrome is an idiosyncratic drug reaction presenting after a latent period of 2-6 weeks from the initiation of offending drugs. DRESS syndrome commonly manifests as rash, hematological abnormalities, lymphadenopathy, and internal organ involvement. Several medications can cause DRESS syndrome, and recognition with prompt discontinuation of such drugs is imperative. Even more important is avoiding the re-exposure of the responsible drug is necessary. Initially, the disease was attributed to phenytoin and was called phenytoin hypersensitivity syndrome [2]. Anti-seizure medications (carbamazepine, phenytoin, phenobarbital, lamotrigine), allopurinol, and sulfonamides are the most common offending drugs in the majority of the cases. A more complete list of medications that are implicated in DRESS syndrome is listed in Table 1 [3].

**Table 1:** Common medications implicated in causation of DRESS syndrome DRESS: Drug reaction with eosinophilia and systemic symptoms; NSAIDs: Nonsteroidal anti-inflammatory drugs.

MEDICATIONS
Anticonvulsants: Carbamazepine, lamotrigine, phenobarbital, phenytoin, valproic acid, and zonisamide
Antibiotics: Ampicillin, cefotaxime, dapsone, quinine, rifampin, ethambutol, isoniazid, trimethoprim-sulfamethoxazole, linezolid, metronidazole, minocycline, pyrazinamide, sulfasalazine, streptomycin, and vancomycin
Antivirals: Abacavir, nevirapine, and zalcitabine
Antidepressants: Bupropion and fluoxetine
Antihypertensives: Amlodipine and captopril
Biologics: Efalizumab and imatinib
NSAIDs: Ibuprofen and Celecoxib
Other: Allopurinol, epoetin alfa, mexiletine, and ranitidine

The incidence of DRESS syndrome is 1 in 1,000 to 10,000 drug exposure [4]. The liver, lungs, and kidneys are commonly affected, manifesting as hepatitis, pneumonitis, and nephritis. Rare, severe and atypical presentations of DRESS syndrome include colonic, endocrine and neurologic involvement [5]. Although eosinophilia is a common presentation, it can be absent in 30-40% of cases, and its appearance may be delayed for several weeks.

The manifestations of DRESS involving the gastrointestinal tract can include nausea, vomiting, bloody, and non-bloody diarrhea. The histopathological manifestations of DRESS can vary from active colitis, as seen in our patient to cryptitis, ulcerations/erosions to eosinophilic infiltration of the lamina propria [5].

A genetic defect in detoxifying enzymes leads to the accumulation of drug metabolites, which in turn bind with cellular proteins and activate T cells. Activated T cells cause cellular death and IL-5 secretion, causing eosinophilia and eosinophilic infiltrates. The possible reactivation of herpes-like virus - HSV-6 and Epstein-Barr virus (EBV) - is also partly responsible for disease manifestations [6]. The clinical features can be very nonspecific, mimicking infectious, neoplastic, or rheumatologic conditions. The differential diagnosis of DRESS syndrome is wide and includes several conditions such as Stevens-Johnson syndrome/toxic epidermal necrolysis (SJS/TEN), acute generalized exanthematous pustulosis (AGEP), hypereosinophilic syndrome (HES), and erythroderma [7]. Due to the inconsistency of symptoms, the European Registry of Severe Cutaneous Adverse Reactions (RegiSCAR) scoring system was developed by Kardaun et al., illustrated in Table 2 [8].

**Table 2:** Registry of Severe Cutaneous Adverse Reactions (RegiSCAR) criteria for the diagnosis of DRESS syndrome Three of the four asterisked (*) criteria required for the diagnosis of DRESS syndrome. DRESS: Drug reaction with eosinophilia and systemic symptoms

Diagnostic Criteria
Hospitalization
Reaction suspected to be drug-related
Acute rash
Fever >38 degrees Celsius *
Enlarged lymph nodes at a minimum of two sites *
Involvement of at least one organ *
Blood count abnormalities *
a) Lymphocytes above or below the normal limits
b) Eosinophils above the laboratory limits
c) Platelets below the laboratory limits

This scoring system is used to assist in helping to classify suspected cases as unlikely, possible, probable, or definite cases.

Treatment for DRESS is aimed at discontinuation of the offending drug. Systemic steroids are usually used to reduce the symptoms of hypersensitivity reactions. The French Society of Dermatology recommends the administration of prednisone (1 mg/kg/day) [9]. However, the appropriate route, duration of use and rapidity of steroid taper is unknown and controversial. The use of steroids, especially the duration remains unclear at this time and remains controversial. Since then, several reports have recommended alternative treatment options, for second-line use. Drugs such as cyclosporine, intravenous immunoglobulins, mycophenolate mofetil, cyclophosphamide, and plasmapheresis have been evaluated as alternatives [10-13]. Since viral reactivation is thought to play a role in the pathogenesis of DRESS syndrome, some experts have recommended the use of antivirals, such as ganciclovir, along with steroid treatment [14]. However, further studies need to be done to evaluate the efficacy of this treatment.

As mentioned earlier, the mortality rate in DRESS syndrome is approximately 10-20%. A majority of the time, the patients succumb to fulminant hepatic failure [15].

## Conclusions

DRESS syndrome is associated with the use of commonly used medications. Lamotrigine is a common medication used not only as an anti-epileptic but also as a mood stabilizer in bipolar disorder. Physicians must have a very high degree of suspicion for DRESS in the setting of suspicious signs and symptoms.
